# Genome-informed structural analysis of polymerase and glycoprotein adaptation in H5N1 clade 2.3.4.4b

**DOI:** 10.4142/jvs.26088

**Published:** 2026-07-14

**Authors:** Sangyi Lee, Seunghye Cho, Hyunbeen Kim, Woojin Shin, Soyoung Lee, Atanas V. Demirev, Jin Il Kim

**Affiliations:** 1 Department of Microbiology, Institute for Viral Diseases, Korea University College of Medicine, Seoul 02841, Korea.; 2 Vaccine Innovation Center, Korea University College of Medicine, Seoul 02841, Korea.; 3 Biosafety Center, Korea University College of Medicine, Seoul 02841, Korea.

**Keywords:** Influenza a virus, H5N1 subtype, host-pathogen interactions, sialic acids, ANP32 proteins, zoonoses

## Abstract

**Importance:**

Understanding the molecular mechanisms driving H5N1 clade 2.3.4.4b is critical for pandemic preparedness.

**Objective:**

To characterize the molecular drivers of viral fitness and mammalian adaptability in recent H5N1 viruses by integrating evolutionary dynamics with structural simulations.

**Methods:**

This study analyzed 2,398 H5Nx genomes (2000–2024) through phylogenetic and selective pressure analyses. HA/NA structures were predicted with AlphaFold 3 and evaluated by AutoDock4 docking, whereas polymerase–ANP32A/B complexes were modeled using template-based methods and their binding free energies were estimated using MM/GBSA. Polymerase–ANP32E complexes were predicted with AlphaFold 3 and similarly evaluated by MM/GBSA. The binding affinities (ΔG) for the sialic acid (SA) receptors and human ANP32 proteins were quantified through molecular mechanics/generalized born surface area calculations.

**Results:**

Clade 2.3.4.4b showed significant antigenic drift in the HA receptor binding site, reducing affinity for *α*2,3-SA and *α*2,6-SA receptors. On the other hand, the emergence of a full-length stalk N1 NA with second sialic acid-binding site mutations (e.g., N366S) compensated for reduced HA affinity by enhancing the NA binding stability. In the polymerase complex, both the PB2-627E/631L variant (−144.00 kcal/mol; unadjusted *p* = 0.0058) and the known mammalian-adaptive 627K/631M variant (−144.67 kcal/mol; unadjusted *p* = 0.0165) showed more favorable predicted human ANP32B binding free energies than the ancestral 627E/631M state (−136.46 kcal/mol).

**Conclusions and Relevance:**

The co-occurrence of HA, NA, PB1, and PB2 signatures was associated with clade expansion and produced structural predictions consistent with altered receptor or ANP32 interactions; experimental validation is required before inferring effects on fitness or zoonotic risk.

## INTRODUCTION

Since the initial emergence of the A/goose/Guangdong/1/96 (Gs/Gd) lineage, highly pathogenic avian influenza (HPAI) A/H5 viruses have disseminated globally via migratory routes of wild birds, posing severe threats to the poultry industry and public health through zoonotic spillover [1]. The evolutionary success of these viruses is driven by molecular adaptations in key viral proteins [2]. Hemagglutinin (HA) has undergone significant diversification into 10 clades, with mutations in the key residues modulating binding affinity and preference toward avian-like α2,3-sialic acid (SA) or human-like α2,6-SA receptors [3]. Similarly, the neuraminidase (NA) gene has exhibited complex dynamics, particularly the reassortment that led to the emergence of the H5N8 clade 2.3.4.4 [1] and its subsequent transition to the currently dominant H5N1 clade 2.3.4.4 [4]. Beyond surface glycoproteins, mutations in the internal polymerase complex (e.g., PB2-E627K and PA-T85A) play critical roles in enhancing viral replication, virulence, and mammalian adaptability [5].

Despite continuous global surveillance providing a wealth of genetic data, characterizing the phenotypic impact of these evolutionary shifts remains a significant challenge. The viral fitness of HPAI A/H5 is not determined by isolated mutations but by the highly entwined and synergistic dynamics among all eight gene segments. Although previous studies have often focused on individual segments or specific mutations, no comprehensive, genome-wide analyses have integrated these multifaceted mutational dynamics to explain why certain lineages, such as the recent clade 2.3.4.4 H5N1, achieve global dominance while others fade. Understanding the correspondence between multi-segment mutational profiles and their collective effects on viral fitness is crucial for accurate risk assessment and pandemic preparedness.

This study conducted a comprehensive genome-informed characterization of recent clade 2.3.4.4 HPAI H5N1 viruses by integrating the evolutionary and mutational dynamics of their complete gene segments. The specific correspondence between the emergence of novel strains carrying key mutations and the temporal shifts in clade abundance was examined. Furthermore, structural reconstruction of conformational and ligand-binding changes was used to determine how these synergistic mutations collectively drive viral fitness and global dominance. This approach provides a holistic framework for understanding the molecular drivers behind the success of current HPAI A/H5 lineages.

## METHODS

### Data collection and alignment

H5 genomes were obtained from the Global Initiative on Sharing All Influenza Data (GISAID) (https://platform.epicov.org/) and the National Center for Biotechnology Information (NCBI) Influenza Virus Resource (https://www.ncbi.nlm.nih.gov/genomes/FLU/) database on May 30, 2025 [6]. Each gene segment was aligned independently. The HA and N1 NA gene segments were aligned to the reference strain A/Goose/Guangdong/1/96 (H5N1) using Nextclade CLI v3.8.2 [7]. The NA gene segments from other subtypes were aligned using MAFFT v7.490 [8] with default parameters. All alignments were manually curated and trimmed to the open reading frame. Sequences containing gaps exceeding 2% of the total alignment length were excluded. Sequences with less than 90% gene coverage were excluded. Redundant and near-identical sequences were removed using CD-HIT v4.8.1 [9] with a 99.98% similarity threshold, generating a non-redundant dataset. Sequences containing more than one amino-acid gap (excluding biologically plausible indels) were also excluded. The final curated dataset of 2,398 H5 virus isolates for mutational analysis throughout history, and the dataset of 650 H5 virus isolates for zoonotic cases, were examined. Specific accession numbers are provided in Supplementary Data 1 and 2.

### Mutational analysis

The 2,398 sequences were used to detect amino acid mutations temporally correlated with relative clade abundance and cross-species infection frequency. Mutations were indicated based on the amino acid residues of a representative H5N2 virus (A/duck/Hebei/2/2011, EPI_ISL_137254). By each gene segment, each strain was classified into the clade using LABEL (v.0.6.3) [10]. The maximum-likelihood phylogenies for each gene segment were estimated by IQ-TREE (v.2.2.3.3) and FastTree (v.2.1.11) [11,12]. Augur (v.23.1.1) [13] suggested an amino acid sequence at each residue from all nodes and tips per week and estimated the weekly relative frequencies of amino acid sequence and viral clades using the Kernel density estimation centered on the sampling date with an interval of 0.05 years. The relative clade abundance per week was calculated by dividing the number of sequences in the clade by the sum of the number of sequences in a week. The weekly frequency of cross-species infection was calculated from the approximate counts of tips labeled as “human” or “non-human mammalian” using the kernel density estimation with an interval of 0.05 years in each molecular clock phylogeny of eight gene segments. The relative frequency of amino acid sequences per residue, the relative clade abundance, and the frequency of cross-species infection were normalized and smoothed by aggregating into a Gaussian distribution. The K-means clustering method identified amino acid mutations with significant cross-correlation coefficients with relative clade abundance and cross-species infection frequency within 6-month rolling windows (*p* value < 0.05). The multivariate correlations in regular time series were estimated using the ‘tslearn’ library on Python (v.3.11) and the ‘RolWinMulCor’ package on R (v.4.4.1) [14,15].

### Detection of key amino acid residues under positive selection and epistasis

The selection pressure per amino acid residue was measured from the sub-lineages using an estimation of the ratio of nonsynonymous (dN) to synonymous (dS) substitutions (dN/dS, also known as ω) using 4 approaches: single-likelihood ancestor counting (SLAC), fixed effects likelihood (FEL) [16], fast unconstrained Bayesian approximation (FUBAR) [17], and mixed-effects model evolution (MEME) [18] implemented in the HyPhy (v.2.5.14) [19] with the cutoffs of *p* value < 0.01 (FEL and MEME), < 0.1 (SLAC), and posterior probability > 0.9 (FUBAR). The selection pressures were also estimated using FEL and MEME for the subset alignments divided by sampling year and regions. The epistasis within and between gene segments was detected using Bayesian graphical models (*p* value < 0.05) [20]. The epistasis between the 2 gene segments was detected using a concatenated alignment of the 2 gene segments, as in the detection of epistasis within a single gene segment.

### Structural modeling and protein-ligand interaction analysis

The protein structures of the HA and NA for clades 2.3.4 and 2.3.4.4 were predicted using the sampled sequences by AlphaFold 3 [21]. The sampled sequences genetically identical to the consensus sequences of the HA and NA genes in the clades 2.3.4 and 2.3.4.4, respectively, were selected from the alignments. The HA sequence sampled from Vietnam in 2005 (EPI270188) and the NA sequence sampled from China in 2007 (EPI141281) were selected for protein structure prediction of HA and NA in clade 2.3.4. The HA sequence sampled from Spain in 2022 (EPI1998410) and the NA sequence sampled from the USA in 2022 (EPI2246583) were selected, as for the protein structure prediction of the HA and NA in clade 2.3.4.4. The structural and binding information of the ligands, the N-acetylneuraminic acid (Neu5Ac) in the 6’ sialyllacto-N-tetraose pentasaccharides (SLN-LC) and the 3’SLN-LC, with the second sialic acid-binding site (2SBS) in the NA, were extracted from the binding structure of the NA N9 subtype (PDB ID: 1MWE [22]). The same information on ligands, 3’SLN-LC and 6’SLN-LC, with the HA extracted from the 2 HA H5 subtypes (PDB IDs: 5AJM [23] and 4CQR [23], respectively). The electric charges on the 2 surface proteins and the ligands were reassigned by adding the polar hydrogen atoms and the Kollman charges. The protein-ligand binding was evaluated in the grid box of 40 × 40 × 40 (Å) centered on the SA molecule. The Lamarckian genetic algorithm was used to generate 100 conformations for each protein-ligand binding and predict the representative binding pose and energy. The protein–ligand binding prediction was conducted using Autodock (v4.2.6) [24] and OpenBabel [25]. Visualization was achieved using the UCSF Chimera [26].

After generating lists of residue mutations using custom Python scripts, protein–ligand binding predictions were performed using the Schrödinger software platform. Residue scanning was conducted against a reference sequence using BioLuminate70 (Schrödinger, LLC, USA) to construct mutant protein structures for each sequence. The HA and NA proteins were combined with SA ligands from reference PDBs via the Glide [27] merging function. Molecular Mechanics/Generalized Born Surface Area (MM/GBSA) binding affinity analyses were then performed using Prime [28], with the OPLS4 force field applied for all calculations.

### Phylogenetic and phylogeographic analysis

Two distinct datasets were prepared for phylogenetic analysis. The first dataset consisted of a representative subset of 500 complete genome sequences selected from the finalized 2,398 sequences to optimize computational feasibility for phylogeographic reconstruction. The second dataset comprised 408 randomly sampled HA and NA gene pairs specifically for analyzing the epistasis between HA and NA in H5N1 viruses. For both datasets, a stratified random subsampling approach was used to ensure the subsets accurately reflected the diversity of the original dataset in terms of host species, geographic sampling regions, and temporal distribution (sampling year).

The phylogenies for both datasets were estimated by Bayesian inference using the Markov chain Monte Carlo (MCMC) Metropolis–Hastings algorithm implemented in the Bayesian Evolutionary Analysis by Sampling Trees (v1.10.4) [29] with the GTR + I + G substitution model, uncorrelated lognormal relaxed clock model, and the Bayesian SkyGrid tree priors [30]. Bayesian stochastic search variable selection (BSSVS) was used to estimate the indicators of the transition rates between each pair of the global regions. The MCMC chain length was initiated with 100 million steps with a 10% burn-in and increased until MCMC chains reached reliable convergence and stationarity [31]. The convergence and stationarity of the MCMC chains were assessed by effective sample sizes (ESSs) and tracer plots in Tracer (v 1.7) [32]. The phylogeny estimation was accepted if the ESSs of all continuous parameters exceeded 100 with well-mixed tracer plots. The maximum clade credibility tree was summarized from using TreeAnnotator (v 1.10.5) and visualized using FigTree (v 1.4.4) [33,34].

For the phylogeographic reconstruction (performed on the 500-sequence dataset), BSSVS were used to estimate the indicator of the transition rates between each pair of global regions. Phylogenetic diffusion analysis was performed to calculate the Bayes factor (BF) and the posterior distributions of indicators specifying the transition rates between each pair of the global regions in the spatial phylogenetic reconstruction of evolutionary dynamics. The BF was used to quantify the statistical support for the transitions between two global regions, and it was considered significant if it exceeded 10.

### Data construction for the ANP32 protein binding affinity calculations

A dataset of 95 H5N1 clade 2.3.4.4b polymerase complex sequences was curated to evaluate the effects of host-specific signatures on binding affinity to human ANP32 proteins. These sequences were sampled randomly from 650 H5N1 clade 2.3.4.4b isolates collected between 2021 and 2025 and available in the GISAID database, with representation from avian, human, and bovine hosts. Sequence selection was further guided by a variation at PB2 residues 627 and 631, two adaptive sites previously identified by Chopra et al. [35].

### *In silico* mutational scanning and binding affinity calculations

For a systematic evaluation of the 95 sequences, polymerase complexes associated with human ANP32 proteins (ANP32A, ANP32B, and ANP32E) were constructed by template-based modeling in Maestro v2024-4 (Schrödinger) [36]. The structural framework for ANP32A and ANP32B was based on the crystal structures of the influenza polymerase complexed with these host factors (PDB ID: 8RMR [37] and 8R1J [38], respectively). Owing to the lack of a crystal structure for human ANP32E, its structure was predicted computationally using AlphaFold3 based on its sequence (NCBI Gene ID: 81611), which yielded a high-confidence prediction score (pTM = 0.92). Mutant protein structures were generated by introducing residue-specific substitutions into the reference scaffold using the residue_scanning_backend.py script [13]. The prime_residue refinement method was applied to ensure accurate conformational sampling of substituted side chains [36]. This method optimizes the side-chain geometry of the mutated residue while keeping the backbone and surrounding environment rigid. The binding affinity was evaluated in fast mode using a comprehensive set of descriptors, including hydropathy, van der Waals surface complementarity, solvent-accessible surface area (total, polar, and nonpolar components), and rotatable bond penalties.

After initial residue scanning, the binding affinity was further quantified using MM/GBSA calculations. These calculations were performed in Prime with the OPLS4 force field to obtain high-accuracy energetic estimates. For each complex, the binding free energy (∆G) was calculated to rank the effects of host-specific mutations on the affinity for the human ANP32 protein interfaces [28]. Unadjusted pairwise *t*-tests were performed using R (v.4.4.1) to assess the statistical significance of the differences in binding affinities among the PB2 variant groups, which were grouped by the combination of residue 627 and 631.

## RESULTS

### Collecting H5Nx genome sets

For mutational analysis, 2,398 HA and NA gene sequences from H5Nx viruses collected globally from avian and mammalian hosts from 2000 to 2024 were analyzed. All selected genomes contained a complete set of eight gene segments with fully documented collection dates. Subsampling was performed by the host species to mitigate sampling bias. Consequently, more than half of the H5Nx sequences originated from poultry (28.3%, 678/2,398), while 59.5% (1,427/2,398) were from wild birds. 6.7% were isolated from mammals, including humans (5.2%, 124/2,398) and non-human mammals (1.5%, 37/2,398). The remaining 5.5% was isolated from unknown sources, such as the environment. The host information was obtained from associated metadata and previous studies [39]. H5 clade classification followed Nextclade (https://clades.nextstrain.org). For clarity, clade 2.3.4.4 was further divided into subclade 2.3.4.4b and a grouped category (2.3.4.4a and 2.3.4.4c-h), designated as 2.3.4.4x clades (Supplementary Table 1).

An additional 650 sequences containing H5 full segments were incorporated for further analyses involving zoonotic cases. The inclusion of these non-avian sequences increased the proportion of mammalian-derived data, including 80 mammalian cases, 38 of which were identified in dairy cows (Supplementary Data 2).

### Continuous antigenic drift in the HA gene

Antigenic drift in the HA gene mainly shapes molecular characteristics of the novel clade 2.3.4.4 H5N1 viruses. The robust evolutionary markers were identified by conducting a comprehensive selection pressure analysis using multiple algorithms (MEME, FEL, SLAC, and FUBAR) across datasets stratified by temporal periods and geographic regions. Through this multi-layered screening, 5 key residues (156, 157, 171, 205, and 239 in H5 numbering) were identified around the receptor binding sites, based on their correlation with relative clade abundance and selection pressure (Fig. 1A, Supplementary Table 2). The antecedent H5N1 clade 2.3.4 had N, K, and S in the residues 171, 205, and 239, respectively, but these were substituted into D, N, and R through the diversification into the current H5N1 clades 2.3.4.4. Residues 156 and 157 showed genetic heterogeneity over the antecedent H5N1 clade 2.3.4 and the H5N2, H5N6, and H5N8 subclades of 2.3.4.4. On the other hand, the current H5N2 and H5N1 subclade 2.3.4.4. primarily harbored 156A and 157P. The conformational changes in the receptor binding domain (RBD) caused the loss of hydrogen bonds with the α2,3-SA and α2,6-SA (Fig. 1B). As a result, the current H5N1 2.3.4.4 had lower predicted free binding energies of the RBD with the α2,3-SA (−2.92 kcal/mol) and α2,6-SA (−1.58 kcal/mol) than the antecedent H5N1 clade 2.3.4 (α2,3-SA: −3.34 kcal/mol, α2,6-SA: −2.01 kcal/mol) (Table 1).

**Fig. 1:**
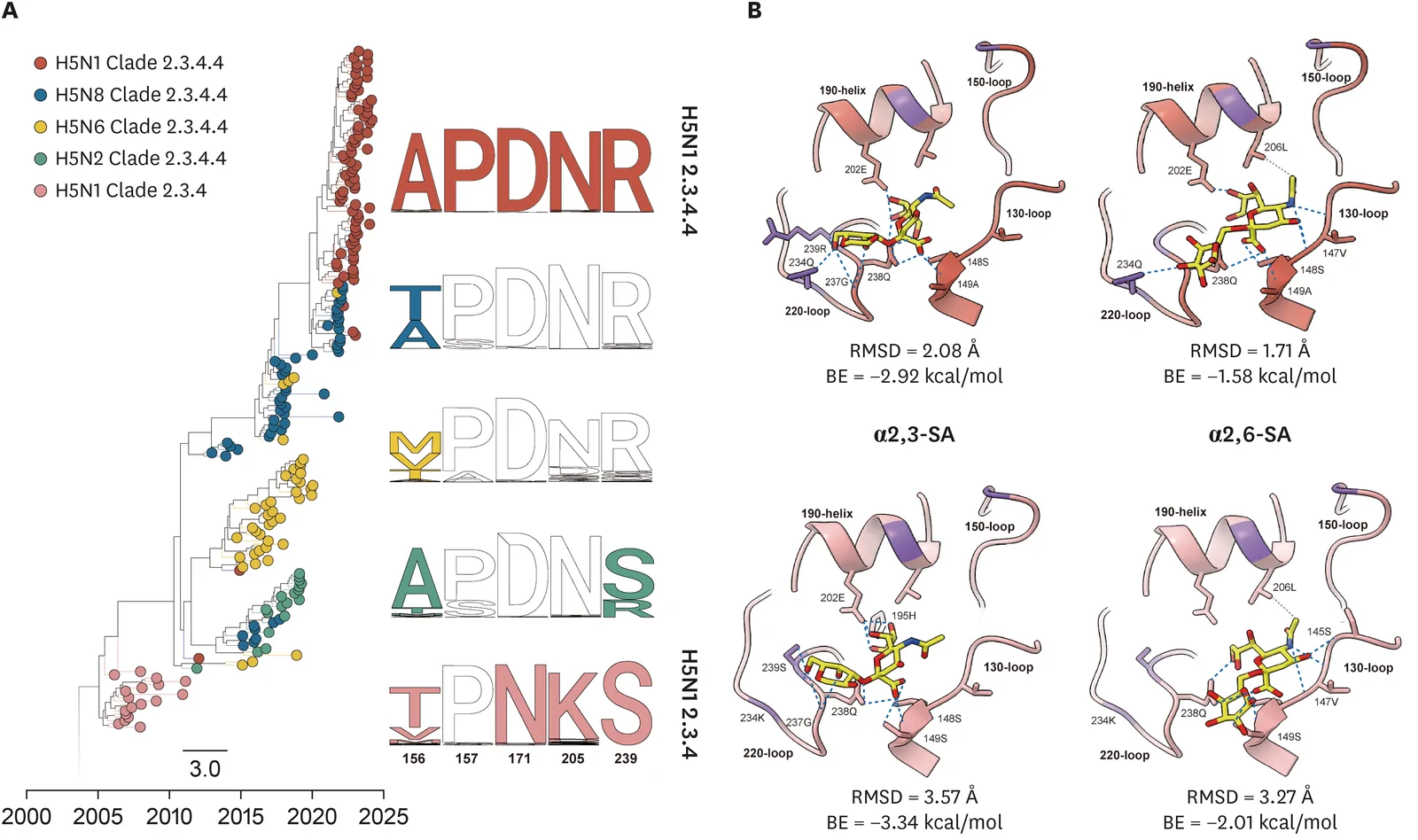
Evolutionary dynamics and structural analysis of the HA gene in H5Nx clades 2.3.4 and 2.3.4.4. (A) Phylogenetic transition and key molecular signatures. The maximum-likelihood phylogeny of the HA gene shows the evolutionary divergence from H5N1 clade 2.3.4 to the highly pathogenic H5Nx clade 2.3.4.4. Representative consensus mutations at the RBS were identified from a total of unique genomic sequences (n = 500) retrieved from Global Initiative on Sharing All Influenza Data and National Center for Biotechnology Information. (B) Structural receptors of the RBS and receptor-binding affinity. Molecular docking simulations were performed to visualize the interactions between representative HA structures from each clade and host cell receptor (α2,3-SA, avian-type; α2,6-SA, human-type). Hydrogen bonds (blue dashed lines) and hydrophobic interactions (grey dashed lines) were calculated using AutoDock (v4.2.6) [24]. All structural metrics represent the highest score with a proper pose for each strain. HA, hemagglutinin; RBS, receptor-binding site; SA, sialic acid; RMSD, root mean square deviation; BE, binding energy.

**Table 1:** Summary of the protein (HA and NA protein)-ligand (SAs) docking simulation

Protein motif	Ligand	HA clades^a^	RMSD (Å)^b^	Binding energy (kcal/mol)	Inhibition constant (Ki) (Temp = 298.15K)^c^	No. of H bonds (protein-SA)^d^	Amino acid involved in interactions
HA RBS	α2,3-SA	2.3.4	3.57	−3.34	3.57 mM	14	148S, 149S, **195H**, 202E, 237G, 238Q, 239S
		2.3.4.4	2.08	−2.92	7.26 mM	11	148S, 149A, 202E, **234Q**, 237G, 238Q, 239R
	α2,6-SA	2.3.4	3.27	−2.01	33.80 mM	7	145S, 147V, 149S, **206L**, 238Q
		2.3.4.4	1.71	−1.58	69.42 mM	8	147V, 148S, 149A, **202E**, **234Q**, 238Q
NA enzymatic sites	α2,3-SA	2.3.4	3.76	−6.74	11.50 µM	5	118R, **295N**, **368R**, 402Y
		2.3.4.4	2.96	−3.76	1.75 mM	2	118R, 402Y
	α2,6-SA	2.3.4	4.11	−7.05	6.02 µM	12	118R, **146N**, **147G**, **148T**, **151D**, **156R**, **179W**, **225R**, **278E**, 402Y
		2.3.4.4	2.90	−6.59	14.69 µM	3	118R, 295N, 402Y
NA 2SBS	α2,3-SA	2.3.4	2.14	−1.05	169.35 mM	7	**364S**, **366N**, 367S, 369S, 399W
		2.3.4.4	2.20	−1.27	117.35 mM	7	367S, 369S, **397T**, 399W, **432K**
	α2,6-SA	2.3.4	2.22	−1.04	173.56 mM	9	**329N**, 364S, 365T, 366N, 367S, **369S**
		2.3.4.4	2.37	−1.60	67.60 mM	8	364S, 365T, 366S, 367S, **396I**, **397T**, **399W**, **432K**

Bold fonts indicate residues involved in binding with SAs that are different between the HA 2.3.4 and 2.3.4.4 clades.

HA, hemagglutinin; NA, neuraminidase; SA, sialic acid; RMSD, root mean square deviation; Ki, inhibition constant (mM, millimolar); RBS, receptor-binding site; 2SBS, second sialic acid-binding site.

^a^HA clades in the NA protein mean the HA clades of the corresponding viruses.

^b^After the comparison with the reference PDB structures, RMSD, which indicates the root-mean-square deviation between two poses of the same ligand molecule docked on the same protein, is determined.

^c^The inhibition constant of the complex formed between the HA or NA protein motif and SA ligands indicates the affinity between the ligand and the protein binding site. A smaller value signifies a stronger affinity.

^d^The number of bonds formed between the protein motif and SAs.

### Full-length stalk N1 NA of the current H5N1 clade 2.3.4.4

The majority of the current H5N1 clade 2.3.4.4 harbored the HA gene, which originated from the H5N8 clade 2.3.4.4, mainly reported in wild birds in Europe (time to the most recent common ancestor (tMRCA): April 30, 2018 [95% highest posterior density {HPD} interval: July 28, 2017–May 14, 2019]). The novel N1 NA gene was predominantly involved in the low pathogenic avian influenza (LPAI) in the non-Gs/Gd lineages, infecting wild birds in Europe (median of tMRCA: February 25, 2017 [95% HPD: July 11, 2016 –June 18, 2018]). In particular, the novel N1 NA gene exhibited a full-length stalk longer than that of the antecedent N1 NA gene (Fig. 2A). In addition, the novel N1 NA gene harbored amino acid mutations in the enzymatic sites and the 2SBS compared to the antecedent N1 NA gene (Fig. 2, Supplementary Fig. 1). The conformational changes imposed by these mutations affected the binding affinity at the enzymatic sites and 2SBS with the α2,3-SA and α2,6-SA. The novel and antecedent N1 NAs had significantly different binding topologies with α2,3-SA from those with α2,6-SA (Fig. 2B). Consequently, the predicted free energies at the enzymatic sites with the α2,3-SA were much lower in the novel N1 NA (−3.76 kcal/mol) than the antecedent N1 NA (−6.74 kcal/mol), but those with α2,6-SA were similar between the novel (−6.59 kcal/mol) and antecedent (−7.05 kcal/mol) N1 NAs (Table 1). Interestingly, the key substitutions in the novel N1 NA, particularly N366S and A395E (N2 numbering), also altered the topology at 2SBS binding with the α2,3-SA and α2,6-SA (Fig. 2B). As a result, the novel N1 NA showed higher predicted binding energies at the 2SBS with the α2,3-SA (−1.27 kcal/mol) and α2,6-SA (−1.6 kcal/mol) than the antecedent N1 NA (α2,3-SA: −6.74, α2,6-SA: −7.59 kcal/mol) (Supplementary Fig. 1).

**Fig. 2:**
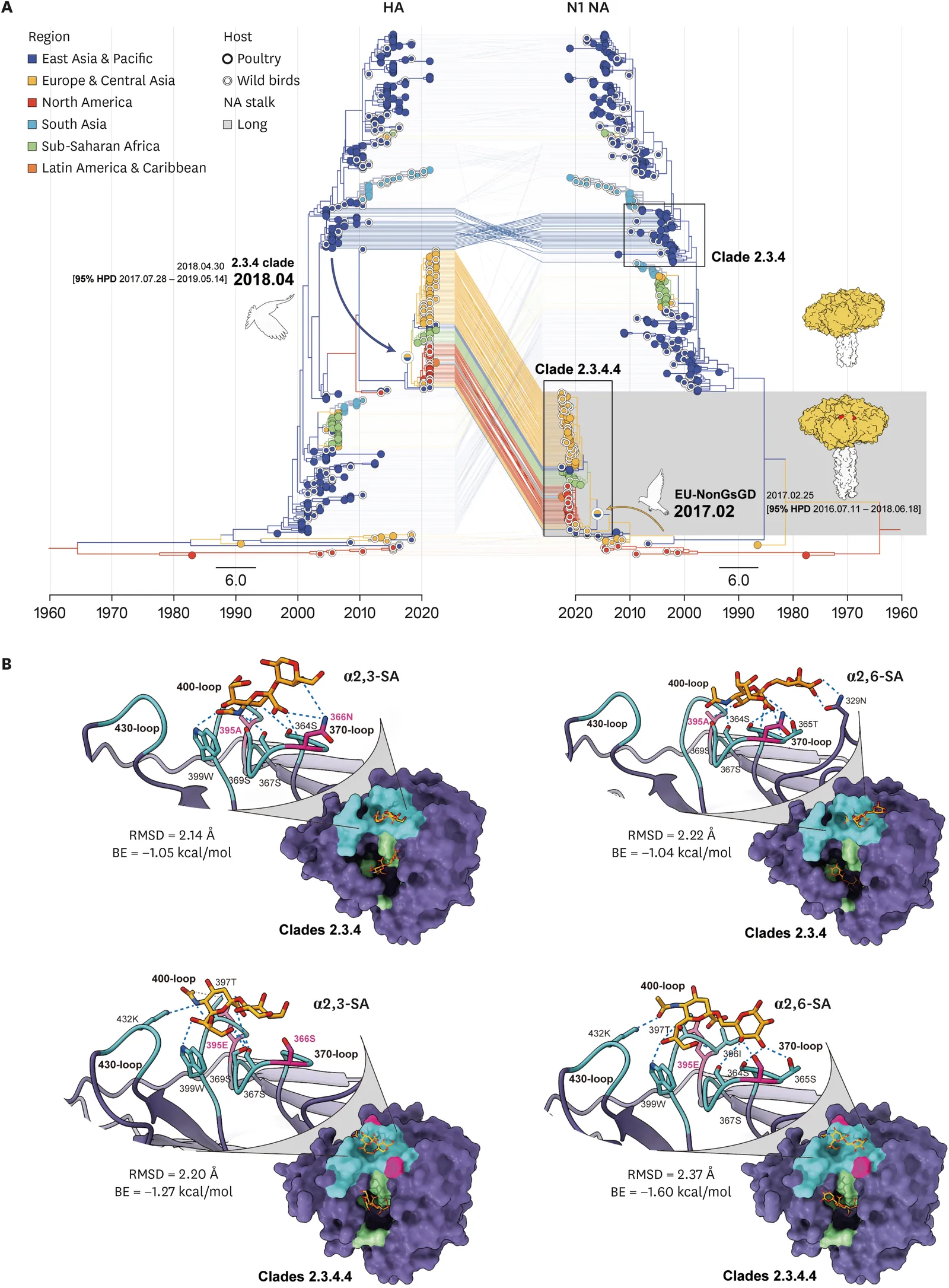
Phylogenetic and structural evolution of the N1 NA gene in H5N1 clades 2.3.4 and 2.3.4.4. (A) Evolutionary co-divergence of HA and NA genes. The tanglegram represents a head-to-head comparison of maximum-likelihood trees for the HA and NA genes. Lines connecting the trees illustrate the reassortment history and co-evolutionary patterns within H5N1 viruses. The black boxes delineate the transition from clade 2.3.4 to 2.3.4.4, highlighting significant structural shifts in the N1 NA protein. Phylogenetic trees were constructed using (n = 408) sequences. (B) Comparative structural analysis of the 2SBS. Detailed structural configurations of the 2SBS are shown for representative H5N1 strains from clade 2.3.4 and clade 2.3.4.4 in complex with avian-type (α2,3-SA) and human (α2,6-SA) receptors. The 2SBS is highlighted in cyan, conserved enzymatic sites in green, and key functional residues at positions 395 and 399 are in magenta. The blue and grey dashed lines denote hydrogen bonds and hydrophobic interactions, respectively, identified via Autodock (v4.2.6) [24]. All structural metrics represent the highest score with a proper pose for each strain. NA, neuraminidase; HA, hemagglutinin; 2SBS, second sialic acid-binding site; SA, sialic acid; RMSD, root mean square deviation; BE, binding energy.

### Molecular dynamics and zoonotic potential of the polymerase complex

All eight segments were screened for mutations correlating with mammalian infection cases to identify genomic drivers of cross-species transmission. Although the evolutionary dynamics varied across segments, the polymerase basic protein 1 (PB1) showed the strongest correlation with temporal surges in mammalian infections. Specifically, the current H5N1 clade 2.3.4.4 harbored two convergent amino acid substitutions, 172D and 430K in the PB1, which coincided with the temporal surge in cross-species infections in mammalian species. In particular, the PB1 clade with only the 430K involved many non-human mammal H5Nx infection sequences. The clade with 172D and 430K involved many human H5Nx infection cases (Fig. 3A).

**Fig. 3:**
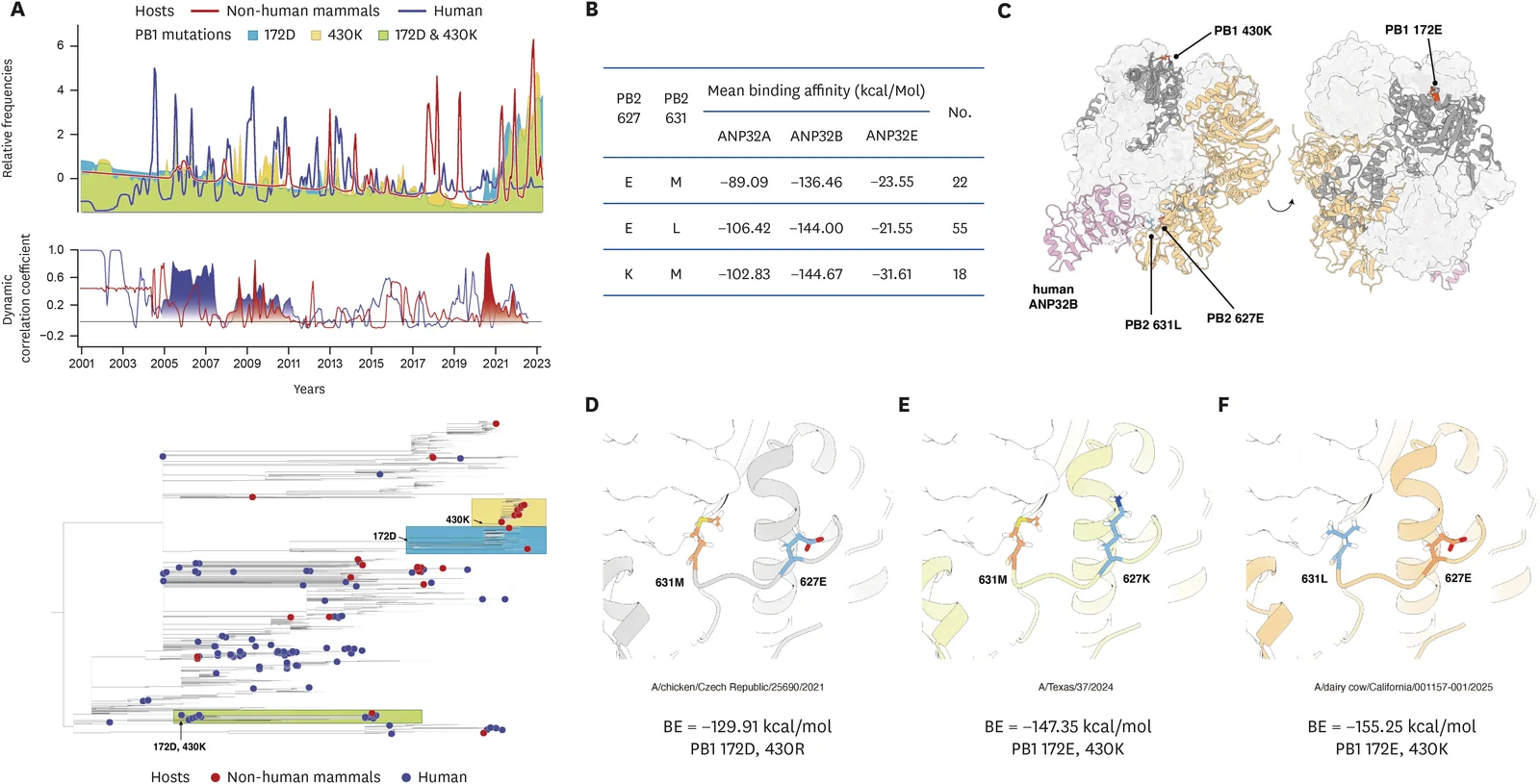
Two key mutations in the PB1 gene related to cross-species infection from avian to mammal. (A) The linear plots display the temporal changes in two key mutations 172D (blue) and 430K (yellow), the co-occurrence of both mutations (green) in the PB1 gene, and the weekly frequencies of non-human mammal and human cases and their dynamic correlation coefficients. (B) The phylogeny of the PB1 gene labeling sequences of non-human mammal (red) and human (blue), and highlighting clades harboring 172D (blue), 430K (yellow) and both mutations (green). (C) The summary results of mean binding affinity of PB2 627/631 variants to human ANP32 proteins, estimated by molecular mechanics/generalized born surface area (kcal/mol). (D) Template-based model of the polymerase-ANP32B complex generated using PDB 8R1J, shown in front and back views, with the back view rotated by 180° relative to the front view. ANP32B is colored pink, PB2 orange, PB1 dark gray, and PA light gray. PB1 residues 172 and 430 are highlighted in orange-red. (D-F) Close-up views of PB2 residues 627 (skyblue) and 631 (light orange) from representative strains of each host: (D) avian (A/chicken/Czech_Republic/25690/2021), (E) human (A/Texas/37/2024), and (F) bovine (A/dairy_cow/California/001157-001/2025). PB, polymerase basic protein; BE, binding energy.

The synergistic effects of PB1 mutations in combination with known PB2 host-adaptive markers (627 and 631) were investigated to move beyond phylogenetic correlation and establish a mechanistic basis for these observations. Structural modeling of the polymerase complex in association with human ANP32A, ANP32B, and ANP32E showed that these mutations collectively modulate viral fitness in mammalian environments. Consistent with previous observations, 627K showed a higher binding affinity than 627E. In particular, the 627E/631L variant showed comparable binding affinity to that of 627K/631M.

For ANP32B, the mean binding affinity values of 627K/631M (−144.67 kcal/mol [95% confidence interval [CI], −150.09, −139.25]) and 627E/631L (−144.00 kcal/mol [95% CI, −146.63, −141.36]) variants, frequently observed in recent bovine H5N1 cases, were stronger than that of the 627E/631M variants (−136.46 kcal/mol [95% CI, −141.86, −131.05]; *p* = 0.0165, and *p* = 0.0058, respectively) (Fig. 3B). This trend was closely mirrored in the ANP32A interactions, where the 627K/631M (−102.83 kcal/mol [95% CI, −112.31, −93.36]) and 627E/631L (−106.42 kcal/mol [95% CI, −111.51, −101.32]) variants exhibited significantly higher binding affinities compared to the 627E/631L (−89.09 kcal/mol [95% CI, −94.90, −83.28]; *p* = 0.0167 and *p* = 0.0002, respectively).

In contrast, interactions with ANP32E generally yielded weaker baseline binding scores across all variants. This overall reduction in calculated affinity was attributed to the lack of a crystal protein structure for ANP32E, which necessitated the use of an AlphaFold 3-predicted model for the structural analyses. Despite these lower baseline scores, the 627K/631M variant (−31.61 kcal/mol [95% CI, −41.14, −22.08]) showed a significantly stronger affinity than the 627E/631L variant (−21.55 kcal/mol [95% CI, −26.11, −16.98]; *p* = 0.041).

In addition, residue-level structural differences were examined using representative strains from each host species, including the overall complex structure (Fig. 3C) and representative avian, human, and bovine strain structures (Fig. 3D-F). Furthermore, when these PB2 signatures were paired with the identified PB1 mutations (172E and 430K), the binding energy for the bovine-derived complex (A/dairy cow/California/001157-001/2025) improved significantly to −155.25 kcal/mol, compared to the avian-like A/chicken/Czech Republic/25690/2021 strain (−129.91 kcal/mol) (Fig. 3D-F). These results suggest that the accumulation of specific mutations across the polymerase subunits (PB1 and PB2) facilitates a more stable interaction with the human ANP32B mediator, potentially bypassing the temperature-dependent restrictions of avian-origin polymerases.

## DISCUSSION

This paper presented the molecular characteristics of the current H5N1 clade 2.3.4.4 in the context of evolutionary dynamics following the divergence from the H5Nx clades. The key genetic features of the current H5N1 clade 2.3.4.4, including relative clade abundance, selection pressure, epistasis, and binding affinity, were observed. Specifically, the current H5N1 clade 2.3.4.4 harbored the key molecular characteristics well-adapted to a wide range of host species in diverse ecologies.

The mutations in the HA genes primarily shaped the molecular characteristics of the current H5N1 clade 2.3.4.4, including the wide range of cross-species infection. HA is a major factor in influencing the host species as well as pathogenicity by the binding affinity with the α2,3-SA and α2,6-SA [40]. The current H5N1 clade 2.3.4.4 showed five key mutations in the HA gene, inducing conformational change in the 130 and 220-loops and the 190-helix of the RBD, resulting in lowered binding affinities with the α2,3-SA and α2,6-SA by reforming the chemical bond links (Fig. 1). In particular, the S239R mutation elucidated the adaptation of H5Nx clade 2.3.4.4 to a broad spectrum of hosts, ranging from domestic poultry to wild avian and mammal species. This residue was identified as a crucial genetic determinant for which mutations can significantly alter the viral binding affinity with the host’s SAs [4]. The presence of serine in this residue was positively selected in domestic poultry in Asian regions, resulting in the dominance of the antecedent H5N1 2.3.4 with a strong binding affinity with SAs (Supplementary Figs. 2 and 3, Supplementary Data 3 and 4). On the other hand, with the transition of the epicenter from Asian domestic poultry to European wild avian species, the H5Nx clade 2.3.4.4 lineage with R239 spread dominantly, possibly due to a lower binding affinity with SAs, compared to S239 (Supplementary Fig. 4) [41]. The mutations in the HA gene possibly facilitated the effective dissemination of the H5Nx clade 2.3.4.4 lineage along the migratory routes of wild migratory birds, promoting moderate virulence in the wild hosts [42].

The elongated stalk and mutations in the 2SBS are likely associated with the current dominance of the current H5N1 clade 2.3.4.4. The antecedent H5N1 clade 2.3.4 was highly virulent and well-adapted in the Asian domestic poultry because of the short-length stalk of the NA. Since the divergence of the subclade 2.3.4.4, the antecedent H5N1 viruses have reassorted with different NA subtypes, N2, N6, and N8, reported in Asian and African wild birds, characterized by elongated stalk regions resulting from the addition of three N-linked glycosylations. The full-length stalk NA reassortment could compensate for the lowered binding affinity of the HA with SAs because the full-length stalk NA could grant higher efficiency of viral budding with enhanced access to SAs on the host receptors [43] accompanied by lower host specificity for crossing the species barrier (Fig. 2, Supplementary Fig. 1) [44]. Furthermore, key mutations in the 2SBS likely may have played a role in the evolutionary success of the current H5N1 subclade 2.3.4.4. The 2SBS is a unique genetic domain of NA of the avian influenza functionally related to catalytic activity [3]. Generally, the genetic sequences of the 2SBS are conserved because the mutations can disrupt the functional balance between HA and NA [45]. The current H5N1 clade 2.3.4.4 exhibited a conformational change in the 2SBS through amino acid substitutions. Specifically, the N366S substitution appeared to increase the binding affinity with α2,3-SA to α2,6-SA by bringing the 430-loop and likely played an important role in the adaptation by compensating for HA binding affinity with SAs in avian and mammalian species.

This study identified specific mutations within the internal genes that may be associated with the better adaptation of the current H5N1 clade 2.3.4.4 across a wide range of host species. The mutations in the PB1 gene, such as the 172D and 430K residues, were correlated with relative clade abundance aligned with past mammalian infection cases observed in H5N1 during the years 2005–2010. Considering previous studies on the genetic heterogeneity by the host species at residue 430 in PB1 [46], the current H5N8 clade 2.3.4.4 might exhibit a higher propensity for cross-species transmission to mammalian species (Fig. 3). The inclusion of PB2 molecular characterization further strengthens the understanding of host-range expansion. The identified mutations in PB2, combined with the aforementioned PB1 residues, may enhance the polymerase activity in mammalian cells, potentially overcoming the temperature-dependent restriction of avian influenza viruses. The selection of three representative sequences reflecting these PB1 and PB2 combinations underscores the non-random accumulation of these mutations, providing a hypothetical basis for an evolutionary trajectory toward increased zoonotic potential (Fig. 3).

Despite the comprehensive assessment of the evolutionary dynamics of all eight gene sequences, molecular characterization of the current H5N1 clade 2.3.4.4 may be affected by irregular sampling intensity. Nevertheless, an attempt was made to mitigate this impact by subsampling genetic sequences by time and location, measuring local selection pressure at each residue, and reviewing previous research to identify key mutations associated with transmissibility and cross-species infection of H5Nx viruses (Supplementary Tables 2 and 3). Thus, the current characterization of the current H5N1 clade 2.3.4.4 sufficiently captures the genetic characteristics of dominant lineages, based on representative evolutionary dynamics of H5Nx viruses.

This study solely evaluated the additive effects of key mutations on the evolutionary dynamics of the H5Nx viruses, but it did not measure the synergistic or antagonistic effects among mutations. The present study monitored the change in the evolutionary dynamics of H5Nx viruses through the emergence of variants with amino acid mutations. Nevertheless, it is difficult to capture the combined effects of key mutations. A better understanding of complex epistatic interactions among the eight gene segments, such as the functional balance between the HA and NA proteins, can help explain the evolutionary dynamics of H5Nx viruses and enable more accurate assessments of the viral fitness of novel variants (Supplementary Fig. 5) [47]. These findings would generally benefit from experimental validation and future in vitro or in vivo studies comparing the recombinant viruses to measure the combined effects of key mutations on viral fitness.

Since the emergence of vaccination of poultry against the antecedent H5N1 clade 2.3.4 in China, the HPAI H5Nx clade 2.3.4.4 lineage suddenly acquired adaptive mutations through antigenic drift and shift [48]. The accumulation of adaptive mutations, coupled with the changes in epicenter, reduced the virulence and specificity of the host range species of H5Nx viruses. Specifically, this paper presented the accumulation of adaptive mutations in the currently dominant HPAI H5N1 clade 2.3.4.4. still preferring moderate virulence and a broad range of hosts, including mammalian species [49]. Fortunately, with the increasing zoonotic potential through mammalian adaptation, the current H5N1 variants do not harbor an oseltamivir-resistant mutation in the N1 NA (e.g., NA-H274Y) [50]. Hence, an emergency antiviral treatment against the zoonotic strain could still be recommended. Nevertheless, vigilant monitoring of the evolution of the H5N1 variants, particularly the acquisition of human-adapted and/or antiviral-resistant mutations, will be a key factor in determining the success of prevention and control measures against the emergence of the next pandemic H5Nx strains.

## SUPPLEMENTARY MATERIALS

Supplementary Data 1 The accession numbers of all the sequences used in the study deposited in GISAID (https://gisaid.org/) and NCBI (https://www.ncbi.nlm.nih.gov/)

Supplementary Data 2 Summary metadata of the H5 sequences used in this study

Supplementary Data 3 Kernel Density Estimation illustrating the Gaussian distributions of H5Nx subclades and concurrent mutations

Supplementary Data 4 Codon-site-level selective pressure tests for episodic diversifying residues (MEME) and pervasive positive or negative selection (FEL)

Supplementary Table 1 Number of sequences for each subclade, host, and region

Supplementary Table 2 Summary results of selective pressure and epistasis for every selected residue

Supplementary Table 3 Previous studies on the selected residues for each segment

Supplementary References

Supplementary Fig. 1 Structures of NA genes illustrating sialic acid binding at the enzymatic sites and 2SBS. This figure highlights the differences in binding conformations of N1 NA from the 2.3.4 and 2.3.4.4 clades to 2-3Sia and 2-6Sia, with a specific focus on the enzymatic sites and the 2SBS region.

Supplementary Fig. 2 Kernel Density Estimation illustrating Gaussian distributions of H5N1 2.3.4.4 subclades and concurrent mutations. The area graphs show the relative frequency of H5N1 2.3.4.4 subclades, and the line graphs show the frequencies of mutations.

Supplementary Fig. 3 Codon-site-level selective pressure tests for episodic diversifying residues (MEME). Results of the annual selective pressure analysis (MEME) for each gene segment are shown. The magnitudes of LRT values are colored in purple according to the top-left corner of the figure legend.

Supplementary Fig. 4 Annual chord diagram of transmission events between regions. Chord diagram illustrating the introduction events between regions resulting from phylogeographical analysis. Chords distant from the edge represent influxes, and the width of the chord is proportional to the number of flows.

Supplementary Fig. 5 Tanglegram illustrating phylogeographical trees for HA and NA genes of H5N1 clades, including information on mutations and epistasis. The Bayesian Evolutionary Analysis by Sampling Trees-derived phylogeographic trees of HA and N1 NA genes display key mutations annotated in text. Epistatic relationships are indicated by dashed grey arrows. The regions and hosts are denoted according to the legend at the top left of the figure.

## References

[1] Demirev AV, Park H, Lee K, Park S, Bae JY, Park MS (2023). Phylodynamics and molecular mutations of the hemagglutinin affecting global transmission and host adaptation of H5Nx viruses. Transbound Emerg Dis.

[2] Pulit-Penaloza JA, Brock N, Pappas C, Sun X, Belser JA, Zeng H (2020). Characterization of highly pathogenic avian influenza H5Nx viruses in the ferret model. Sci Rep.

[3] Liu L, Chen G, Huang S, Wen F (2023). Receptor binding properties of neuraminidase for influenza a virus: an overview of recent research advances. Virulence.

[4] Huang P, Sun L, Li J, Wu Q, Rezaei N, Jiang S (2023). Potential cross-species transmission of highly pathogenic avian influenza H5 subtype (HPAI H5) viruses to humans calls for the development of H5-specific and universal influenza vaccines. Cell Discov.

[5] Herfst S, Schrauwen EJ, Linster M, Chutinimitkul S, de Wit E, Munster VJ (2012). Airborne transmission of influenza A/H5N1 virus between ferrets. Science.

[6] Shu Y, McCauley J (2017). GISAID: global initiative on sharing all influenza data - from vision to reality. Euro Surveill.

[7] Aksamentov I, Roemer C, Hodcroft EB, Neher RA (2021). Nextclade: clade assignment, mutation calling and quality control for viral genomes. J Open Source Softw.

[8] Katoh K, Standley DM (2013). MAFFT multiple sequence alignment software version 7: improvements in performance and usability. Mol Biol Evol.

[9] Fu L, Niu B, Zhu Z, Wu S, Li W (2012). CD-HIT: accelerated for clustering the next-generation sequencing data. Bioinformatics.

[10] Shepard SS, Davis CT, Bahl J, Rivailler P, York IA, Donis RO (2014). LABEL: fast and accurate lineage assignment with assessment of H5N1 and H9N2 influenza A hemagglutinins. PLoS One.

[11] Price MN, Dehal PS, Arkin AP (2010). FastTree 2--approximately maximum-likelihood trees for large alignments. PLoS One.

[12] Minh BQ, Schmidt HA, Chernomor O, Schrempf D, Woodhams MD, von Haeseler A (2020). IQ-TREE 2: new models and efficient methods for phylogenetic inference in the genomic era. Mol Biol Evol.

[13] Huddleston J, Hadfield J, Sibley TR, Lee J, Fay K, Ilcisin M (2021). Augur: a bioinformatics toolkit for phylogenetic analyses of human pathogens. J Open Source Softw.

[14] Tavenard R, Faouzi J, Vandewiele G, Divo F, Androz G, Holtz C (2020). Tslearn, a machine learning toolkit for time series data. J Mach Learn Res.

[15] Polanco-Martínez JM (2020). RolWinMulCor: an R package for estimating rolling window multiple correlation in ecological time series. Ecol Inform.

[16] Pond SL, Frost SD (2005). Datamonkey: rapid detection of selective pressure on individual sites of codon alignments. Bioinformatics.

[17] Murrell B, Moola S, Mabona A, Weighill T, Sheward D, Kosakovsky Pond SL (2013). FUBAR: a fast, unconstrained bayesian approximation for inferring selection. Mol Biol Evol.

[18] Murrell B, Wertheim JO, Moola S, Weighill T, Scheffler K, Kosakovsky Pond SL (2012). Detecting individual sites subject to episodic diversifying selection. PLoS Genet.

[19] Kosakovsky Pond SL, Poon AFY, Velazquez R, Weaver S, Hepler NL, Murrell B (2020). HyPhy 2.5-a customizable platform for evolutionary hypothesis testing using phylogenies. Mol Biol Evol.

[20] Avino M, Poon AFY (2019). Detecting amino acid coevolution with Bayesian graphical models. Methods Mol Biol.

[21] Abramson J, Adler J, Dunger J, Evans R, Green T, Pritzel A (2024). Accurate structure prediction of biomolecular interactions with AlphaFold 3. Nature.

[22] Varghese JN, Colman PM, van Donkelaar A, Blick TJ, Sahasrabudhe A, McKimm-Breschkin JL (1997). Structural evidence for a second sialic acid binding site in avian influenza virus neuraminidases. Proc Natl Acad Sci U S A.

[23] Xiong X, Xiao H, Martin SR, Coombs PJ, Liu J, Collins PJ (2014). Enhanced human receptor binding by H5 haemagglutinins. Virology.

[24] Morris GM, Huey R, Lindstrom W, Sanner MF, Belew RK, Goodsell DS (2009). AutoDock4 and AutoDockTools4: automated docking with selective receptor flexibility. J Comput Chem.

[25] O’Boyle NM, Banck M, James CA, Morley C, Vandermeersch T, Hutchison GR (2011). Open babel: an open chemical toolbox. J Cheminform.

[26] Pettersen EF, Goddard TD, Huang CC, Couch GS, Greenblatt DM, Meng EC (2004). UCSF Chimera--a visualization system for exploratory research and analysis. J Comput Chem.

[27] Friesner RA, Banks JL, Murphy RB, Halgren TA, Klicic JJ, Mainz DT (2004). Glide: a new approach for rapid, accurate docking and scoring. 1. Method and assessment of docking accuracy. J Med Chem.

[28] Jacobson MP, Pincus DL, Rapp CS, Day TJ, Honig B, Shaw DE (2004). A hierarchical approach to all-atom protein loop prediction. Proteins.

[29] Drummond AJ, Suchard MA, Xie D, Rambaut A (2012). Bayesian phylogenetics with BEAUti and the BEAST 1.7. Mol Biol Evol.

[30] Gill MS, Lemey P, Faria NR, Rambaut A, Shapiro B, Suchard MA (2013). Improving Bayesian population dynamics inference: a coalescent-based model for multiple loci. Mol Biol Evol.

[31] Lemey P, Rambaut A, Drummond AJ, Suchard MA (2009). Bayesian phylogeography finds its roots. PLOS Comput Biol.

[32] Rambaut A, Drummond AJ, Xie D, Baele G, Suchard MA (2018). Posterior summarization in Bayesian phylogenetics using tracer 1.7. Syst Biol.

[33] Baele G, Carvalho LM, Brusselmans M, Dudas G, Ji X, McCrone JT (2025). HIPSTR: highest independent posterior subtree reconstruction in TreeAnnotator X. Bioinformatics.

[34] Rambaut A (2018). FigTree v1. 4.4.

[35] Chopra P, Page CK, Shepard JD, Ray SD, Kandeil A, Jeevan T Receptor binding specificity of a bovine A(H5N1) influenza virus. bioRxiv.

[36] (2024). Schrödinger Release 2024-4: Maestro.

[37] Arragain B, Krischuns T, Pelosse M, Drncova P, Blackledge M, Naffakh N (2024). Structures of influenza A and B replication complexes give insight into avian to human host adaptation and reveal a role of ANP32 as an electrostatic chaperone for the apo-polymerase. Nat Commun.

[38] Staller E, Carrique L, Swann OC, Fan H, Keown JR, Sheppard CM (2024). Structures of H5N1 influenza polymerase with ANP32B reveal mechanisms of genome replication and host adaptation. Nat Commun.

[39] Xie R, Edwards KM, Wille M, Wei X, Wong SS, Zanin M (2023). The episodic resurgence of highly pathogenic avian influenza H5 virus. Nature.

[40] Stencel-Baerenwald JE, Reiss K, Reiter DM, Stehle T, Dermody TS (2014). The sweet spot: defining virus-sialic acid interactions. Nat Rev Microbiol.

[41] Guo H, de Vries E, McBride R, Dekkers J, Peng W, Bouwman KM (2017). Highly pathogenic influenza A(H5Nx) viruses with altered H5 receptor-binding specificity. Emerg Infect Dis.

[42] Claes F, Morzaria SP, Donis RO (2016). Emergence and dissemination of clade 2.3.4.4 H5Nx influenza viruses-how is the Asian HPAI H5 lineage maintained. Curr Opin Virol.

[43] McAuley JL, Gilbertson BP, Trifkovic S, Brown LE, McKimm-Breschkin JL (2019). Influenza virus neuraminidase structure and functions. Front Microbiol.

[44] Castrucci MR, Kawaoka Y (1993). Biologic importance of neuraminidase stalk length in influenza A virus. J Virol.

[45] Du W, Wolfert MA, Peeters B, van Kuppeveld FJM, Boons GJ, de Vries E (2020). Mutation of the second sialic acid-binding site of influenza A virus neuraminidase drives compensatory mutations in hemagglutinin. PLoS Pathog.

[46] Waters K, Wan HJ, Han L, Xue J, Ykema M, Tao YJ (2021). Variations outside the conserved motifs of PB1 catalytic active site may affect replication efficiency of the RNP complex of influenza A virus. Virology.

[47] Lyons DM, Lauring AS (2018). Mutation and epistasis in influenza virus evolution. Viruses.

[48] Li J, Gu M, Liu K, Gao R, Sun W, Liu D (2020). Amino acid substitutions in antigenic region B of hemagglutinin play a critical role in the antigenic drift of subclade 2.3.4.4 highly pathogenic H5NX influenza viruses. Transbound Emerg Dis.

[49] Long JS, Mistry B, Haslam SM, Barclay WS (2019). Host and viral determinants of influenza A virus species specificity. Nat Rev Microbiol.

[50] Hauge SH, Dudman S, Borgen K, Lackenby A, Hungnes O (2009). Oseltamivir-resistant influenza viruses A (H1N1), Norway, 2007-08. Emerg Infect Dis.

