# Supplementary material for: Genome-informed structural analysis of polymerase and glycoprotein adaptation in H5N1 clade 2.3.4.4b
Source: J Vet Sci. 2026 Jul 14;27(5):e65. doi: 10.4142/jvs.26088 (PMC13655574; doi:10.4142/jvs.26088)
Supplement: Supplementary file 1 [file jvs-27-e65-s003.doc]

**Supplementary Data 3.** Kernel Density Estimation illustrating the Gaussian distributions of H5Nx subclades and concurrent mutations

(A)

# Past H5N1 H5Nx

**H5Nx and H5N1 2.3.4.4 clade**

−3

−2

−1

0

1

2

Dynamic correlation coefficient

−1.0

−0.6

−0.2

0.2

0.6

1.0

0.0

0.2

0.4

0.6

0.8

1.0

P−v

alue

Frequencies of mutations

Time

2023

2019

2015

2013

2011

2009

2007

2001

2021

2017

2005

2003

HA 157P

HA 197S

HA 338L

−1

0

1

2

Dynamic correlation coefficient

−1.0

−0.6

−0.2

0.2

0.6

1.0

0.0

0.2

0.4

0.6

0.8

1.0

P−v

alue

HA 239S

Frequencies of mutations

Time

2023

2019

2015

2013

2011

2009

2007

2001

2021

2017

2005

2003

−3

−2

−1

0

1

2

Dynamic correlation coefficient

−1.0

−0.6

−0.2

0.2

0.6

1.0

0.0

0.2

0.4

0.6

0.8

1.0

Frequencies of mutations

Time

2023

2019

2015

2013

2011

2009

2007

2001

2021

2017

2005

2003

P−v

alue

HA 111L

HA 131L

HA 156A

HA 171D

HA 185R

HA 205N

HA 208K

HA 289H

(B)

# H5N2 H5N6

−1

0

1

2

3

4

Dynamic correlation coefficient

−1.0

−0.6

−0.2

0.2

0.6

1.0

0.0

0.2

0.4

0.6

0.8

1.0

P−v

alue

PA 538V

M2 50Y

Time

2023

2019

2015

2013

2011

2009

2007

2001

2021

2017

2005

2003

Frequencies of mutations

−1

0

1

2

3

4

Dynamic correlation coefficient

−1.0

−0.6

−0.2

0.2

0.6

1.0

0.0

0.2

0.4

0.6

0.8

1.0

P−v

alue

HA 548I

PB2 451L

NP 105I

NP 430A

NS1 92E

NS1 127S

Frequencies of mutations

Time

2023

2019

2015

2013

2011

2009

2007

2001

2021

2017

2005

2003

HA 88S

−1

0

1

2

3

4

Dynamic correlation coefficient

−1.0

−0.6

−0.2

0.2

0.6

1.0

0.0

0.2

0.4

0.6

0.8

1.0

P−v

alue

Time

2023

2019

2015

2013

2011

2009

2007

2001

2021

2017

2005

2003

Frequencies of mutations

# (C)

# H5N2/H5N6

**Cr**

−1

0

1

2

3

4

Dynamic correlation coefficient

−1.0

−0.6

−0.2

0.2

0.6

1.0

0.0

0.2

0.4

0.6

0.8

1.0

P−v

alue

HA 10V

HA 130T

HA 178M

HA 285M

HA 384R

Frequencies of mutations

Time

2023

2019

2015

2013

2011

2009

2007

2001

2021

2017

2005

2003

# (D)

# H5N8

 **H5N8/H5N1**

−1

0

1

2

3

4

Dynamic correlation coefficient

−1.0

−0.6

−0.2

0.2

0.6

1.0

0.0

0.2

0.4

0.6

0.8

1.0

P−v

alue

PB2 292V

Frequencies of mutations

−2

−1

0

1

2

3

4

Dynamic correlation coefficient

−1.0

−0.6

−0.2

0.2

0.6

1.0

0.0

0.2

0.4

0.6

0.8

1.0

P−v

alue

NS1 6M

NS1 17Y

NS1 86S

NS1 143A

NS1 166F

NS1 205N

NS1 213S

NS1 219L

Frequencies of mutations

−3

−2

−1

0

1

2

Dynamic correlation coefficient

−0.6

−0.2

0.2

0.6

1.0

0.0

0.2

0.4

0.6

0.8

1.0

P−v

alue

HA 3N

HA 178I

HA 341K

N1 76A

Time

2023

2019

2015

2013

2011

2009

2007

2001

2021

2017

2005

2003

Frequencies of mutations
