# Supplementary material for: Genome-informed structural analysis of polymerase and glycoprotein adaptation in H5N1 clade 2.3.4.4b
Source: J Vet Sci. 2026 Jul 14;27(5):e65. doi: 10.4142/jvs.26088 (PMC13655574; doi:10.4142/jvs.26088)
Supplement: Supplementary file 2 [file jvs-27-e65-s004.doc]

**Supplementary Data 4.** Codon-site-level selective pressure tests for episodic diversifying residues (MEME) and pervasive positive or negative selection (FEL)

**Annual & Regional FEL & MEME results**

**Note**	*p < 0.1; **p < 0.05; ***p < 0.01.

LRT

15

10

5

0

# Annual FEL results for selected residues

## PB2

451

*******

*******

******

292

## HA

## NP

105 430

******

## N1 NA

405

******

366

10

**2005	2006	2007	2008	2009	2010	2011	2012	2013	2014	2015	2016	2020	2021	2022	2023**

## M2

18	*****	**	**	**	**	**	**	****

## NS1

127

86

166

205

213

**~2000**

**2006**

**2007**

**2008**

**2009**

**2010**

**2011**

**2012**

**2013**

**2014**

**2015**

**2016**

**2020**

**2021**

**2022**

**2023**

**2001~2005**

**2017**

**2018**

**2019**

*******

*******

*******

*******

*******

******

******

******

******

******

******

******

******

******

# Regional FEL results for selected residues PB2

451

**PB1**

172

88

156

178

338

205

548

208

**HA**

171

**M2**

12

18

105

*******

*******

*******

*******

*******

*******

*******

*******

******

******

******

******

******

*******

*******

******

******

**NP**

## NS1

### 219 213

205	*****	****

*******

*******

*******

******

******

******

Asia	EU	North Africa	North America	South Africa	South America	South Asia

#	_PB2_	Regional MEME results for selected residues

**FEL - All annual results**

## PB2

## M1

# FEL - All regional results

## PB2

451

**PB1**

87

154

205

156

548

171

**HA**

153

338

548

**NA N1**

188

16

## NA N6

343

Asia	EU	North Africa	South Africa	South Asia

## M2

20

66

10

14

1

8

12

13

## NS1

213

227

230

216

226

197

210

212

205

209

185

## NEP

26

34

14

Asia	EU	North Africa	North America	South Africa	South America	South Asia

**MEME - All annual results**

## PB2

## M1

| Asia |  | EU |  | North Africa |  | North America |  | South Africa | South America | South Asia |
| --- | --- | --- | --- | --- | --- | --- | --- | --- | --- | --- |
|  |  |  |  |  |  |  |  |  |  |  |
|  |  |  |  |  |  |  |  |  |  |  |
|  |  |  |  |  |  |  |  |  |  |  |
|  |  |  |  |  |  |  |  |  |  |  |
|  |  |  |  |  |  |  |  |  |  |  |
|  |  |  |  |  |  |  |  |  |  |  |
|  |  |  |  |  |  |  |  |  |  |  |
|  |  |  |  |  |  |  |  |  |  |  |
|  |  |  |  |  |  |  |  |  |  |  |
|  |  |  |  |  |  |  |  |  |  |  |
| Asia |  |  | EU |  |  | North Africa |  | South Africa |  | South Asia |
|  |  |  |  |  |  |  |  |  |  |  |
| Asia |  |  | EU |  |  | North America |  | South Amercia |  | South Asia |
|  |  |  |  |  |  |  |  |  |  |  |
|  |  |  |  |  |  |  |  |  |  |  |

## N1 NA

470 367 355 313 188 74 49 42 37 35 16

## N2 NA

392 82

## N6 NA

342

220

152

Asia	EU	North Africa	South Africa	South Asia

### N8 NA

469 468 390

Asia	EU	North Africa	South Africa	South Asia

### M2
