# Supplementary material for: Genome-informed structural analysis of polymerase and glycoprotein adaptation in H5N1 clade 2.3.4.4b
Source: J Vet Sci. 2026 Jul 14;27(5):e65. doi: 10.4142/jvs.26088 (PMC13655574; doi:10.4142/jvs.26088)
Supplement: Supplementary file 3 [file jvs-27-e65-s008.doc]

**Supplementary References**

S1. Huang P, Sun L, Li J, Wu Q, Rezaei N, Jiang S, et al. Potential cross-species transmission of highly pathogenic avian influenza H5 subtype (HPAI H5) viruses to humans calls for the development of H5-specific and universal influenza vaccines. *Cell Discov*. 2023;9(1):58.

S2. Bai R, Sikkema RS, Li CR, Munnink BBO, Wu J, Zou L, et al. Antigenic variation of avian influenza A(H5N6) viruses, Guangdong Province, China, 2014–2018. *Emerg Infect Dis*. 2019;25(10):1932-1945.

S3. Li J, Gu M, Liu K, Gao R, Sun W, Liu D, et al. Amino acid substitutions in antigenic region B of hemagglutinin play a critical role in the antigenic drift of subclade 2.3.4.4 highly pathogenic H5NX influenza viruses. *Transbound Emerg Dis*. 2020;67(1):263-275.

S4. Gao R, Gu M, Shi L, Liu K, Li X, Wang X, et al. N-linked glycosylation at site 158 of the HA protein of H5N6 highly pathogenic avian influenza virus is important for viral biological properties and host immune responses. *Vet Res*. 2021;52(1):8.

S5. Peng W, Bouwman KM, McBride R, Grant OC, Woods RJ, Verheije MH, et al. Enhanced human-type receptor binding by ferret-transmissible H5N1 with a K193T mutation. *J Virol*. 2018;92(10):e02016-e02017.

S6. Cattoli G, Milani A, Temperton N, Zecchin B, Buratin A, Molesti E, et al. Antigenic drift in H5N1 avian influenza virus in poultry is driven by mutations in major antigenic sites of the hemagglutinin molecule analogous to those for human influenza virus. *J Virol*. 2011;85(17):8718-8724.

S7. Peng Q, Zhu R, Wang X, Shi H, Bellefleur M, Wang S, et al. Impact of the variations in potential glycosylation sites of the hemagglutinin of H9N2 influenza virus. *Virus Genes*. 2019;55(2):182-190.

S8. Leon PE, He W, Mullarkey CE, Bailey MJ, Miller MS, Krammer F, et al. Optimal activation of Fc-mediated effector functions by influenza virus hemagglutinin antibodies requires two points of contact. *Proc Natl Acad Sci U S A*. 2016;113(40):E5944-E5951.

S9. He X, Zhou J, Bartlam M, Zhang R, Ma J, Lou Z, et al. Crystal structure of the polymerase PA(C)-PB1(N) complex from an avian influenza H5N1 virus. *Nature*. 2008;454(7208):1123-1126.

S10. Manzoor R, Igarashi M, Takada A. Influenza A virus M2 protein: roles from ingress to egress. *Int J Mol Sci*. 2017;18(12):2649.

S11. Tada T, Suzuki K, Sakurai Y, Kubo M, Okada H, Itoh T, et al. NP body domain and PB2 contribute to increased virulence of H5N1 highly pathogenic avian influenza viruses in chickens. *J Virol*. 2011;85(4):1834-1846.

S12. Ng AK, Zhang H, Tan K, Li Z, Liu JH, Chan PK, et al. Structure of the influenza virus A H5N1 nucleoprotein: implications for RNA binding, oligomerization, and vaccine design. *FASEB J*. 2008;22(10):3638-3647.

S13. Li M, Wang B. Homology modeling and examination of the effect of the D92E mutation on the H5N1 nonstructural protein NS1 effector domain. *J Mol Model*. 2007;13(12):1237-1244.

S14. Li W, Wang G, Zhang H, Xin G, Zhang D, Zeng J, et al. Effects of NS1 variants of H5N1 influenza virus on interferon induction, TNFalpha response and p53 activity. *Cell Mol Immunol*. 2010;7(3):235-242.

S15. Rosário-Ferreira N, Preto AJ, Melo R, Moreira IS, Brito RMM. The central role of non-structural protein 1 (NS1) in influenza biology and infection. *Int J Mol Sci*. 2020;21(4):1511.

S16. Min JY, Li S, Sen GC, Krug RM. A site on the influenza A virus NS1 protein mediates both inhibition of PKR activation and temporal regulation of viral RNA synthesis. *Virology*. 2007;363(1):236-243.

S17. Xiao C, Ma W, Sun N, Huang L, Li Y, Zeng Z, et al. PB2-588 V promotes the mammalian adaptation of H10N8, H7N9 and H9N2 avian influenza viruses. *Sci Rep*. 2016;6(1):19474.

S18. Li Y, Yamakita Y, Krug RM. Regulation of a nuclear export signal by an adjacent inhibitory sequence: the effector domain of the influenza virus NS1 protein. *Proc Natl Acad Sci U S A*. 1998;95(9):4864-4869.

S19. Patil A, Anhlan D, Ferrando V, Mecate-Zambrano A, Mellmann A, Wixler V, et al. Phosphorylation of influenza A virus NS1 at serine 205 mediates its viral polymerase-enhancing function. *J Virol*. 2021;95(6):e02369-e20.

S20. Hale BG, Randall RE, Ortín J, Jackson D. The multifunctional NS1 protein of influenza A viruses. *J Gen Virol*. 2008;89(Pt 10):2359-2376.

S21. Xu K, Klenk C, Liu B, Keiner B, Cheng J, Zheng BJ, et al. Modification of nonstructural protein 1 of influenza A virus by SUMO1. *J Virol*. 2011;85(2):1086-1098.

S22. Zhao N, Sebastiano V, Moshkina N, Mena N, Hultquist J, Jimenez-Morales D, et al. Influenza virus infection causes global RNAPII termination defects. *Nat Struct Mol Biol*. 2018;25(9):885-893.

S23. Schmidt N, Domingues P, Golebiowski F, Patzina C, Tatham MH, Hay RT, et al. An influenza virus-triggered SUMO switch orchestrates co-opted endogenous retroviruses to stimulate host antiviral immunity. *Proc Natl Acad Sci U S A*. 2019;116(35):17399-17408.

S24. Lamotte LA, Tafforeau L. How influenza A virus NS1 deals with the ubiquitin system to evade innate immunity. *Viruses*. 2021;13(11):2309.

S25. Trapp S, Soubieux D, Marty H, Esnault E, Hoffmann TW, Chandenier M, et al. Shortening the unstructured, interdomain region of the non-structural protein NS1 of an avian H1N1 influenza virus increases its replication and pathogenicity in chickens. *J Gen Virol*. 2014;95(Pt 6):1233-1243.

S26. Carrillo B, Choi JM, Bornholdt ZA, Sankaran B, Rice AP, Prasad BV. The influenza A virus protein NS1 displays structural polymorphism. *J Virol*. 2014;88(8):4113-4122.

S27. Blake ME, Kleinpeter AB, Jureka AS, Petit CM. Structural investigations of interactions between the influenza a virus NS1 and host cellular proteins. *Viruses*. 2023;15(10):2063.

S28. Kanrai P, Mostafa A, Madhugiri R, Lechner M, Wilk E, Schughart K, et al. Identification of specific residues in avian influenza A virus NS1 that enhance viral replication and pathogenicity in mammalian systems. *J Gen Virol*. 2016;97(9):2135-2148.

S29. Obayashi E, Yoshida H, Kawai F, Shibayama N, Kawaguchi A, Nagata K, et al. The structural basis for an essential subunit interaction in influenza virus RNA polymerase. *Nature*. 2008;454(7208):1127-1131.

S30. Zhang Y, Cui P, Shi J, Chen Y, Zeng X, Jiang Y, et al. Key amino acid residues that determine the antigenic properties of highly pathogenic H5 influenza viruses bearing the clade 2.3.4.4 hemagglutinin gene. *Viruses*. 2023;15(11):2249.

S31. Antigua KJC, Baek YH, Choi WS, Jeong JH, Kim EH, Oh S, et al. Multiple HA substitutions in highly pathogenic avian influenza H5Nx viruses contributed to the change in the NA subtype preference. *Virulence*. 2022;13(1):990-1004.

S32. Masuda H, Takashi S, Yoshikuni S, Goh H, Kohji M, Daisei M, et al. Substitution of amino acid residue in influenza A virus hemagglutinin affects recognition of sialyl-oligosaccharides containing *N*-glycolylneuraminic acid. *FEBS Lett*. 1999;464(1-2):71-74.

S33. Chambers BS, Li Y, Hodinka RL, Hensley SE. Recent H3N2 influenza virus clinical isolates rapidly acquire hemagglutinin or neuraminidase mutations when propagated for antigenic analyses. *J Virol*. 2014;88(18):10986-10989.

S34. Neumann G, Macken CA, Kawaoka Y. Identification of amino acid changes that may have been critical for the genesis of A(H7N9) influenza viruses. *J Virol*. 2014;88(9):4877-4896.

S35. Chiang C, Chen G, Shih S. Mutations at alternative 5′ splice sites of M1 mRNA negatively affect influenza A virus viability and growth rate. *J Virol*. 2008;82(21):10873-10886.

S36. Lee RTC, Chang HH, Russell CA, Lipsitch M, Maurer-Stroh S. Influenza A hemagglutinin passage bias sites and host specificity mutations. *Cells*. 2019;8(9):958.

S37. Weltman JK, Skowron G, Loriot GB. Influenza A H5N1 hemagglutinin cleavable signal sequence substitutions. *Biochem Biophys Res Commun*. 2007;352(1):177-180.

S38. Sun Y, Tan Y, Wei K, Sun H, Shi Y, Pu J, et al. Amino acid 316 of hemagglutinin and the neuraminidase stalk length influence virulence of H9N2 influenza virus in chickens and mice. *J Virol*. 2013;87(5):2963-2968.

S39. Kajiwara N, Nomura N, Ukaji M, Yamamoto N, Kohara M, Yasui F, et al. Cell-penetrating peptide-mediated cell entry of H5N1 highly pathogenic avian influenza virus. *Sci Rep*. 2020;10:18008.
